# A Study on the Performance of a Silicon Photodiode Sensor for a Particle Dosimeter and Spectrometer

**DOI:** 10.3390/s21238029

**Published:** 2021-12-01

**Authors:** Bobae Kim, Uk-Won Nam, Sunghwan Kim, Sukwon Youn, Won-Kee Park, Jongdae Sohn, Hong Joo Kim, Seh-Wook Lee, Junga Hwang, Sung-Joon Ye, Insoo Jun, Young-Jun Choi

**Affiliations:** 1 Department of Physics, Kyungpook National University, Daegu 41566, Korea; bokim@cern.ch (B.K.); hongjoo@knu.ac.kr (H.J.K.); sehwook.lee@knu.ac.kr (S.-W.L.); 2 Space Science Division, Korea Astronomy and Space Science Institute, Daejeon 34055, Korea; wkpark@kasi.re.kr (W.-K.P.); jdsohn@kasi.re.kr (J.S.); jahwang@kasi.re.kr (J.H.); yjchoi@kasi.re.kr (Y.-J.C.); 3 Department of Radiology, Cheongju University, Cheongju-si 28497, Korea; kimsh@cju.ac.kr; 4 Department of Applied Bioengineering, Graduate School of Convergence Science and Technology, Seoul National University, Seoul 08826, Korea; grasshoppe5@snu.ac.kr (S.Y.); sye@snu.ac.kr (S.-J.Y.); 5 Department of Astronomy and Space Science, University of Science and Technology, Daejeon 34113, Korea; 6 Jet Propulsion Laboratory, California Institute of Technology, Pasadena, CA 91109, USA; insoo.jun@jpl.nasa.gov

**Keywords:** silicon photodiode sensor, proton spectrometer, gamma rays, radiation dosimeter

## Abstract

A lunar vehicle radiation dosimeter (LVRAD) has been proposed for studying the radiation environment on the lunar surface and evaluating its impact on human health. The LVRAD payload comprises four systems: a particle dosimeter and spectrometer (PDS), a tissue-equivalent dosimeter, a fast neutron spectrometer, and an epithermal neutron spectrometer. A silicon photodiode sensor with compact readout electronics was proposed for the PDS. The PDS system aims to measure protons with 10–100 MeV of energy and assess dose in the lunar space environment. The manufactured silicon photodiode sensor has an effective area of 20 mm × 20 mm and thickness of 650 μm; the electronics consist of an amplifier, analog pulse processor, and a 12-bit analog-to-digital converter for signal readout. We studied the responses of silicon sensors which were manufactured with self-made electronics to gamma rays with a wide range of energies and proton beams.

## 1. Introduction

**Figure 1:**
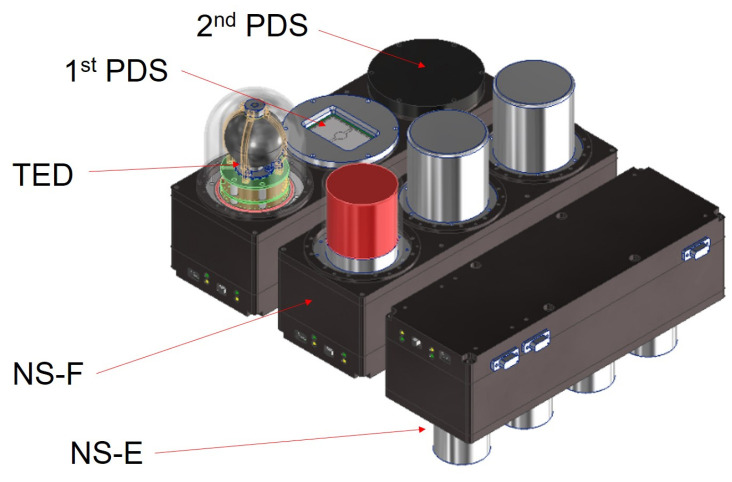
Conceptual drawing of the lunar vehicle radiation dosimeter: a particle dosimeter and spectrometer (PDS), a tissue-equivalent dosimeter (TED), a fast neutron spectrometer (NS-F), and an epithermal neutron spectrometer (NS-E).

The two primary sources of energetic particles on the lunar surface are galactic cosmic rays and solar energetic particles. The lunar vehicle radiation dosimeter (LVRAD) of the Korea Astronomy and Space Science Institute in Korea has been proposed to study the radiation environment on the lunar surface and evaluate its impact on human health. The proposed LVRAD payload is composed of four systems: a particle dosimeter and spectrometer (PDS), a tissue-equivalent dosimeter (TED), a fast neutron spectrometer (NS-F), and an epithermal neutron spectrometer (NS-E), as shown in Figure 1. A silicon photodiode sensor with compact readout electronics was proposed for the PDS. The first and second PDS modules operate in two gain modes and a single gain mode, respectively. We aimed to measure the proton energy range of 10–100 MeV with the silicon detector at the International Space Station.

**Figure 2:**
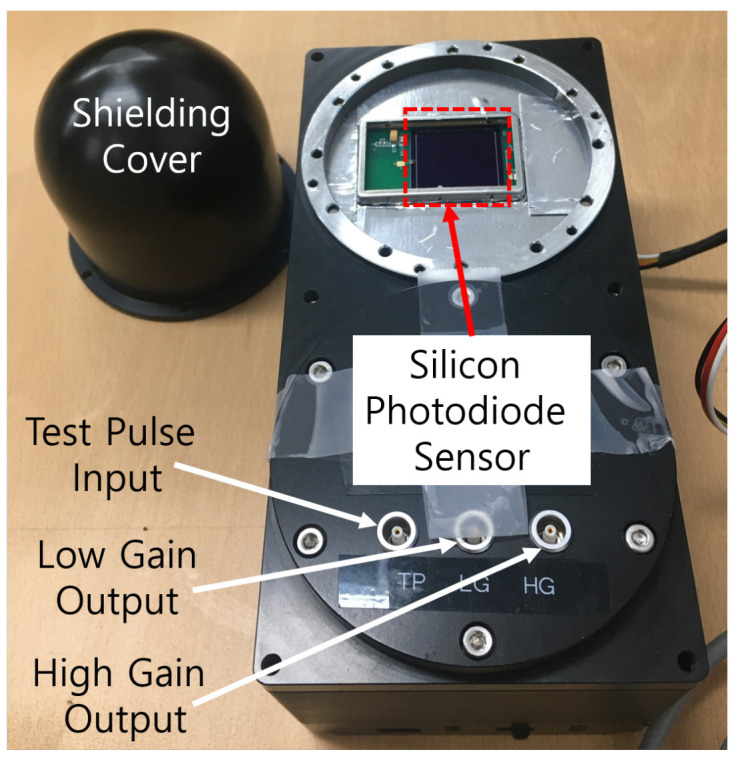
Photograph of a silicon particle dosimeter and spectrometer (PDS).

The mobile dosimeter unit in the portable dosimeter (Liulin-4J) developed by Yukio Uchihori, etc. [1] includes a silicon PIN sensor with an effective area of 10 mm × 20 mm and 300 μm thickness. As particles traverse the material, they slow down and cause the increase in the particle cross-section to stop. The thicker the sensor is, the more energy charged particles can be detected. We manufactured silicon photodiode sensors with 650 μm thickness and developed readout electronics for the PDS system in Figure 2.

In this study, we focused on the responses of the silicon photodiode sensors and readout electronics to gamma rays and proton beams.

## 2. Fabricated Sensor

**Figure 3:**
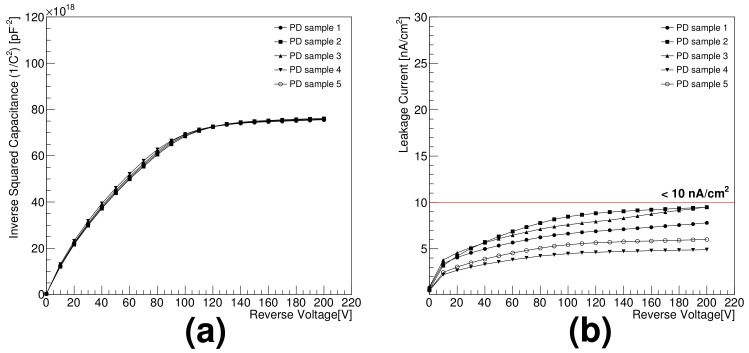
Electrical characteristics of manufactured photodiodes as functions of reverse bias voltage: (**a**) inverse squared capacitance and (**b**) leakage current.

A silicon photodiode sensor with an effective area of 20 mm × 20 mm and 650 ± 30 μm thickness was developed to separate electrons from protons by measuring their energy shower shapes with the silicon detector at the International Space Station [2]. The photodiodes are fabricated on an n-type, high-resistivity (>5 kΩ· cm), double-sided, polished silicon wafer with a 6-in. diameter and <100>-orientation. The conventional double-sided planar fabrication process is performed at the Electronics and Telecommunications Research Institute in Korea. The fabrication steps involve oxidation, photolithography, ion implantation, aluminum sputtering, and passivation. The fabricated sensor can also identify alpha particles because of its light entrance window in the ohmic side of the sensor, which makes it sensitive to incoming particles with short absorption lengths. The sensor design and fabrication process are described in detail elsewhere [3,4]. The capacitance and leakage current of the photodiode sensor are measured with an HP4277A LCZ meter and a Keithley 6517A pico-ammeter, respectively. The inverse distribution of the squared capacitance ($1/C^{2}$) in Figure 3a confirms that the manufactured sensors are fully depleted at approximately 150 V. The measured capacitance is approximately 90 pF at an operation voltage of 200 V. The leakage currents of the sensors have been found to be less than 10 nA/cm${}^{2}$ at the operation voltage, as shown in Figure 3b.

## 3. Developed Electronics

**Figure 4:**
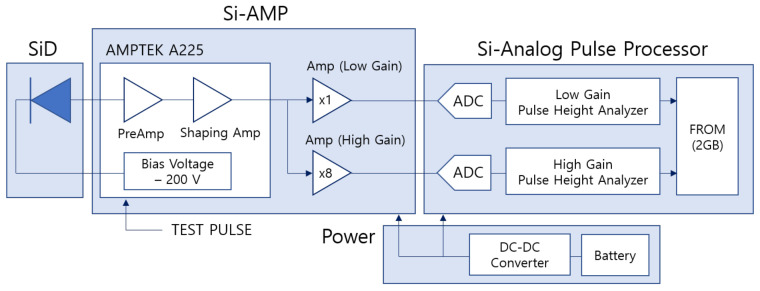
Schematic of the self-made electronics for the particle dosimeter and spectrometer (PDS).

The electronics developed for the signal readout consist of a Si amplifier (Si-Amp), a Si analog pulse processor, and a rechargeable battery, as shown in Figure 4. The Si-Amp consists of an AMPTEK A225 [5], a preamplifier and shaping amplifier, and two amplifiers depending on the low and high gains. The high and low gain modes are used to measure the energies of the incoming gamma rays and charged particles by measuring the energies deposited in the silicon detector, respectively. The signal from the silicon photodiode sensor (SiD) is amplified with 5.2 V/pC and 2.4 μs peaking time; the amplified signal is then shaped by a shaping amplifier. Subsequently, two amplifiers for high gain and low gain amplify the signal, respectively. In the Si analog pulse processor, the analog signals are digitized by the 12-bit analog-to-digital converter; the digitized data are stored as histograms in a flash read-only memory (FROM). The spectrum has 512 ADCs, and the signal voltage corresponds to maximally 2.5 V. Moreover, a rechargeable battery’s output can be converted to the required power through a DC–DC converter, providing proper power for each board. The PDS communicates with a personal computer through RS-422, and we read the data histogram stored in the PDS.

## 4. Experimental Results

**Figure 5:**
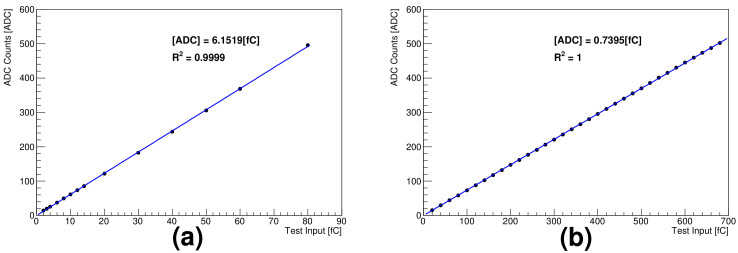
Linearity between test pulse and ADC: (**a**) high gain and (**b**) low gain.

As the high gain is approximately eight times the low gain, charge injection for the linearity test in the high and low gain modes was performed with a test pulse (Figure 5a,b, respectively). The results show good linearity for the low gain mode; however, there was a slight difference in the responses between high and low charge injection ranges in the high gain, as shown in Figure 5a. Therefore, calibration constants in the high gain mode for low- and medium-energy gamma rays with fully deposited energy in the silicon detector were separately obtained. The energies of high-energy gamma rays with a Compton edge were obtained by positioning the Compton edge in the pulse height distribution; in addition, they are compared with the simulation results.

### 4.1. High Gain Mode: Gamma Spectrometer

**Figure 6:**
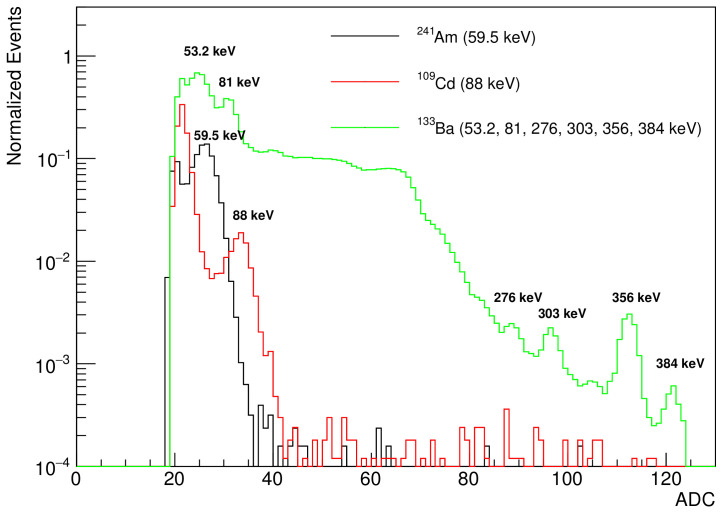
Measured pulse height spectra in high gain mode with a set of low-and medium-energy gamma rays from radioactive gamma sources.

To measure the sensitivity of the SiD as a gamma spectrometer in the high gain mode, gamma rays from the following radioactive sources were used: ${}^{241}\mathrm{Am}$, ${}^{109}\mathrm{Cd}$, and ${}^{133}\mathrm{Ba}$. Figure 6 presents the pulse height spectra of the low-energy gammas from ${}^{133}\mathrm{Ba}$ (53.2 and 81 keV), ${}^{241}\mathrm{Am}$ (59.5 keV), and ${}^{109}\mathrm{Cd}$ (88 keV); and of the medium-energy gammas from ${}^{133}\mathrm{Ba}$ (276, 303, 356, and 384 keV).

**Figure 7:**
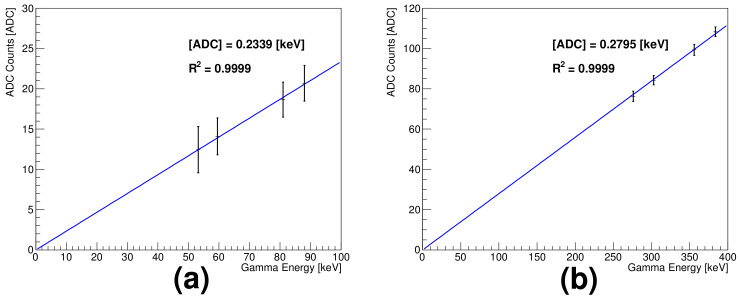
Linearity between measured ADC counts and low and medium-energy gammas: (**a**) 53.2, 59.5, 81, and 88 keV and (**b**) 276, 303, 356, and 384 keV.

The mean ADC value of each full peak was obtained based on the Gaussian function; Figure 7a,b show the measured ADC values of the low- and medium-energy gammas as functions of the gamma energy, respectively.

The results show good linearity; the respective calibration constant is 0.2339 and 0.2795 ADC/keV. The low- and medium-energy gammas, in detector responses, exhibit an approximately 20% difference, which can be explained based on the results from the charge injection tests in the high gain mode. To calibrate the low-energy gammas, ${}^{241}\mathrm{Am}$ is often used; the respective constant 0.2370 ADC/keV is consistent with the linearity measurement results (1.3% deviation). For the medium-energy gammas, the mean ADC value corresponding to 356 keV gammas in the pulse height distribution was used for calibration (it has a calibration constant 0.2791 ADC/keV). This agrees well with the calibration constant from the linearity relationship.

**Figure 8:**
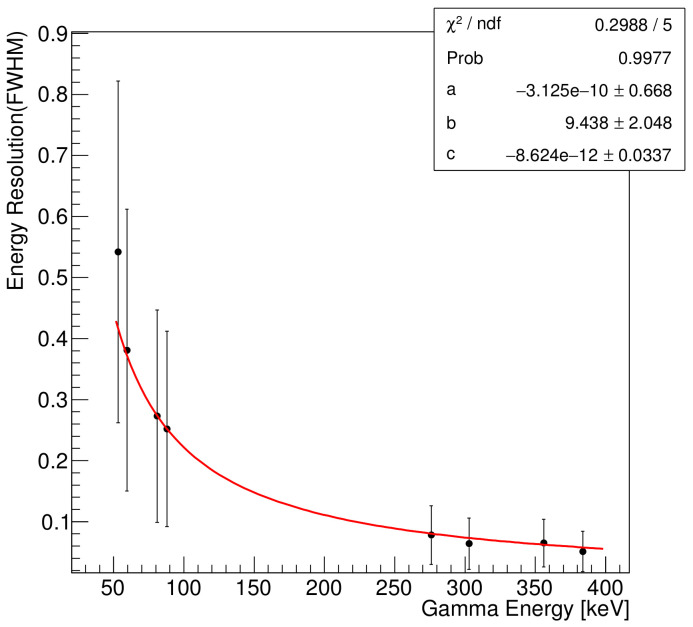
Dependence of energy resolution on gamma energy from radioactive sources: ${}^{241}\mathrm{Am}$, ${}^{109}\mathrm{Cd}$, and ${}^{133}\mathrm{Ba}$.

In general, the energy resolution ($E_{R}$) is as follows [6]:

(1) $$E_{R}=\frac{\sigma (E)}{E}=\frac{a}{\sqrt{E}}⊕\frac{b}{E}⊕c,$$

where the symbol ⊕ denotes a quadratic sum. The first and second terms on the right-hand side of the equation represent the stochastic fluctuation and noise, respectively [6,7]. The third term is a constant. The relative importance of the terms depends on the energy of the incident particle. Figure 8 presents $E_{R}$ as a function of the gamma energy, fitted with the equation for the energy resolution.

The energy resolution improves with increasing gamma energy. The stochastic, noise, and constant terms were determined to be $\left(3.1\times 10^{-8}\right)$%, 9.4 keV, and $\left(8.6\times 10^{-10}\right)$%, respectively. We confirm that the noise term due to the noises of the readout electronics and silicon detector is an important factor in the low- and medium-energy gamma ranges.

The signal to noise ratio (SNR) for low- and medium-energy gammas is calculated as follows:

(2) $$SNR=\frac{Mean_{raw\;signal}-Mean_{pedestal}}{\sigma_{pedestal}},$$

where the signal mean is obtained by subtracting the pedestal mean from the raw signal mean. The energy resolutions and SNRs are summarized in Table 1 and are improved as gamma energies increase [8].

**Figure 9:**
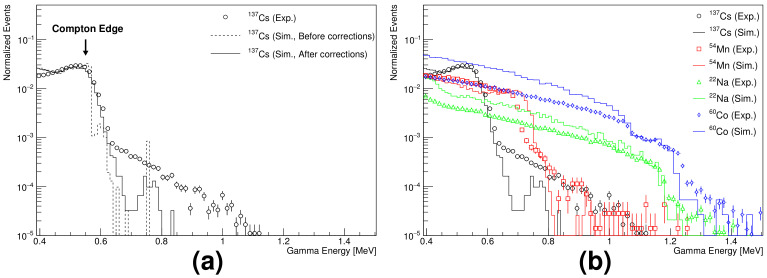
Pulse height distributions of the Compton edge: (**a**) before and after corrections in the simulation, and data for ${}^{137}\mathrm{Cs}$. (**b**) symbols and solid curves represent experimental results and corrected simulation results, respectively.

The position of the Compton edge of the high-energy gammas in the range between 600 keV and 1.5 MeV (from ${}^{137}\mathrm{Cs}$, ${}^{54}\mathrm{Mn}$, ${}^{22}\mathrm{Na}$, and ${}^{60}\mathrm{Co}$ radioactive sources) was determined to be 90% of the maximum pulse height in the pulse height distribution [9,10]. There was a difference between the measurement results and simulation results with the ${}^{137}\mathrm{Cs}$ source. To match these results with GEANT4 [11] simulation results for ${}^{137}\mathrm{Cs}$, 90 keV energy was added; in addition, the energy was smeared according to the energy resolution obtained at 662 keV from Figure 8. After these corrections in the simulation for ${}^{137}\mathrm{Cs}$, the position of the Compton edge agreed well between the measurement and simulation, as shown in Figure 9a. The simulation results of the gammas were calibrated in the same way with higher energies from ${}^{54}\mathrm{Mn}$, ${}^{22}\mathrm{Na}$, and ${}^{60}\mathrm{Co}$ radioactive sources. Figure 9b shows that the Compton edges of the measured high-energy gammas agree well with those of the simulation results within the statistics.

### 4.2. Low Gain Mode: Proton Spectrometer

The proton beam generated in the MC50 cyclotron at the Korea Institute of Radiological and Medical Sciences in Seoul, Korea [12], provides 30 and 45 MeV proton energies. When charged particles pass through a material, they lose their energies owing to ionization, which can be described with the Bethe–Bloch equation [13]. The energy loss rate as a function of the distance through the material reaches its maximum just before the charged particle stops.

**Figure 10:**
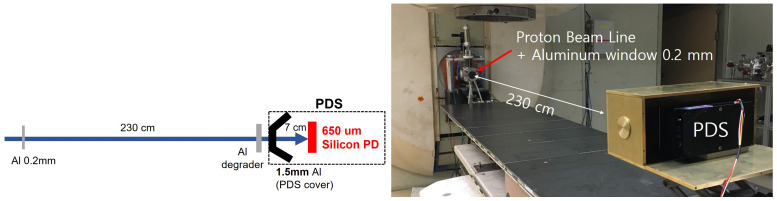
Schematic diagram and picture of an experimental setup for the proton-beam energy measurement in the MC50 cyclotron at the Korea Institute of Radiological and Medical Sciences in Seoul.

In this experiment, the energy of the incident proton beam was reduced with aluminum (Al) metals; the SiD responses to the different proton beam energies were measured in the low gain mode with the same electronics used as for the gamma energy measurements. The proton beam enters the SiD after passing through a 0.2 mm thick Al layer, an Al degrader 230 cm from the Al layer, and a 1.5 mm thick Al cover, and traveling another 7 cm, as shown in Figure 10.

**Figure 11:**
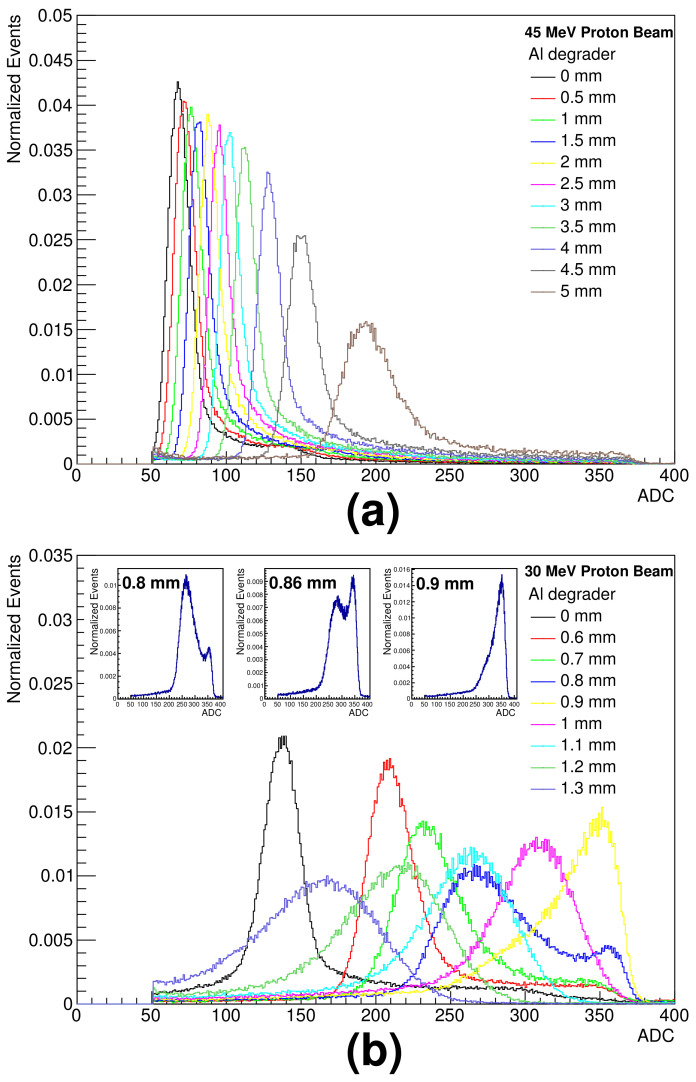
Pulse height distributions of silicon detector for different proton energies obtained with aluminum degraders: (**a**) 45 MeV and (**b**) 30 MeV proton beam energies.

The incident proton beam energies of 30 and 45 MeV decreased to 16.2 MeV and 36.1 MeV at the front of the SiD, respectively, after passing through the geometrical experimental setup in Figure 10. The deposition energies in the SiD for 45 MeV energy were measured by increasing the thickness of the Al degrader from 0.5 to 5 mm in 0.5 mm steps; Figure 11a shows the pulse height distributions for different proton energies.

The energies in the SiD for 30 MeV energy were measured by increasing the thickness of the Al degrader from 0.6 to 1.3 mm in 0.1 mm steps; the pulse height distributions for different proton energies are shown in Figure 11b. The results show that the thicker the degrader, the larger the pulse height and the wider the spectrum. The insets in Figure 11b show the pulse height distributions for 0.8, 0.86 and 0.9 mm thick Al degraders, respectively. The pulse height distributions of the 0.8 and 0.86 mm thick Al degraders are due to the broad incoming proton energy distribution in which the energy below the energy that causes the Bragg peak becomes transferred to the detector. Moreover, some of the higher energy escapes undetected. We expect full energy deposition of the incoming proton energy for Al degraders thicker than 0.9 mm.

**Figure 12:**
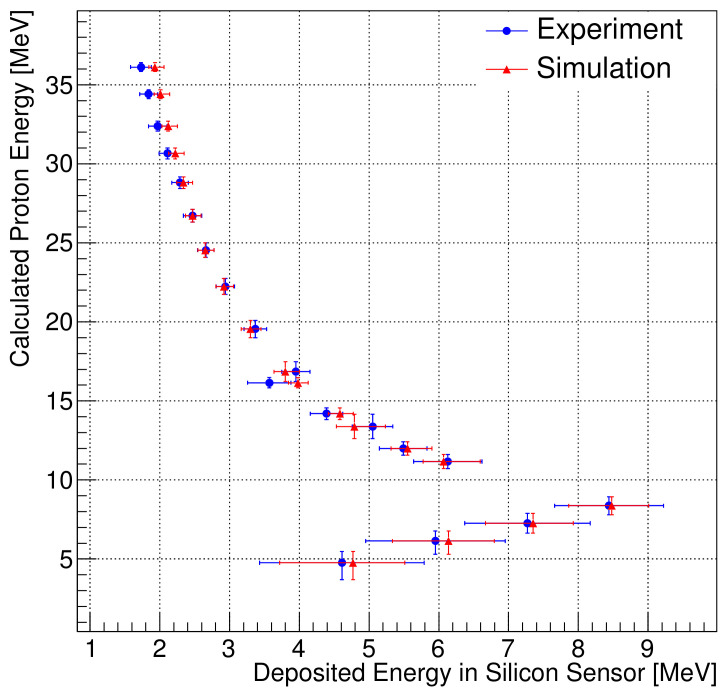
Relationship between deposited energy in the silicon detector and expected energy according to Monte Carlo simulation.

We fit each pulse height distribution with a Gaussian curve; the resulting calibration constant is 1/36.8 MeV/ADC according to the linear fit of the proton energies at the SiD of the GEANT4 simulation and SiD measurement results. The measured and simulated energies are shown in Figure 12; they exhibit a maximal deviation of 4.3%. The results show that the deposited energy in the SiD provides sufficient information about the irradiated proton energies.

## 5. Summary

The manufactured silicon photodiode sensor with a thicker and larger active area and the developed readout electronics that work in the high gain mode properly respond to low gamma energy [${}^{133}\mathrm{Ba}$ (53.2 and 81 keV), ${}^{241}\mathrm{Am}$ (59.5 keV), ${}^{109}\mathrm{Cd}$ (88 keV)], and medium gamma energy (${}^{133}\mathrm{Ba}$ (276, 303, 356, and 384 keV)), which have full energy deposition in the silicon detector. For high-energy gamma rays, which exhibit a Compton edge in the pulse height distribution, the measured energies do not agree well with the expected energies for Compton electrons in the simulation. When corrections based on the ${}^{137}\mathrm{Cs}$ pulse height distribution and proper energy smearing based on energy resolution distribution are applied to the higher-energy gamma rays from ${}^{54}\mathrm{Mn}$, ${}^{22}\mathrm{Na}$, and ${}^{60}\mathrm{Co}$ radioactive sources, the measured Compton edges agree well with those of the simulated Compton electrons. According to the full-width at half-maximum information of the fit spectra, 9.4 keV of the energy resolution is dominated by the noise term due to noise from the readout electronics and silicon detector. The measurement results of the 30 and 45 MeV proton beams show that the incoming proton energy at the silicon detector was measured with 4.3% accuracy or greater. The silicon detector can measure up to 30 MeV proton energy when it operates in the low gain mode. Thus, the fabricated silicon sensors with self-made readout electronics respond properly to a wide range of gamma energies in the high gain mode and proton energies in the low gain mode.

## Figures and Tables

**Table 1:** Energy resolutions and signal to noise ratios for radioactive sources: ${}^{241}\mathrm{Am}$, ${}^{109}\mathrm{Cd}$, and ${}^{133}\mathrm{Ba}$.

Source	Gamma Energy [keV]	Pedestal Mean [ADC]	Pedestal Sigma [ADC]	Signal Mean [ADC]	Signal Sigma [ADC]	$E_{R}$ (FWHM)	SNR
${}^{133}\mathrm{Ba}$	53.2	12.14	1.6	12.5	2.9	0.55 ± 0.28	7.8
${}^{241}\mathrm{Am}$	59.5	11.75	1.1	14.1	2.3	0.38 ± 0.23	12.9
${}^{133}\mathrm{Ba}$	81	12.14	1.6	18.7	2.2	0.28 ± 0.18	11.7
${}^{109}\mathrm{Cd}$	88	12.28	1.6	20.7	2.2	0.25 ± 0.16	12.9
${}^{133}\mathrm{Ba}$	276	12.14	1.6	76.3	2.5	0.08 ± 0.05	47.7
${}^{133}\mathrm{Ba}$	303	12.14	1.6	84.3	2.3	0.06 ± 0.04	52.7
${}^{133}\mathrm{Ba}$	356	12.14	1.6	99.4	2.7	0.06 ± 0.04	62.1
${}^{133}\mathrm{Ba}$	384	12.14	1.6	108.4	2.4	0.05 ± 0.03	67.8

## References

[1] Uchihori Y., Kitamura H., Fujitaka K., Dachev T.P., Tomov B.T., Dimitrov P.G., Matviichuk Y. (2002). Analysis of the calibration results obtained with Liulin-4J spectrometer–dosimeter on protons and heavy ions. Radiat. Meas..

[2] Hyun H.J., Anderson T., Angelaszek D., Baek S.J., Copley M., Coutu S., Han J.H., Huh H.G., Hwang Y.S., Im S. (2015). Performances of photodiode detectors for top and bottom counting detectors of ISS-CREAM experiment. Nucl. Instrum. Methods Phys. Res. Sect. A Accel. Spectrometers Detect. Assoc. Equip..

[3] Jeon H.B., Kang K.H., Park H., Hyun H.J. (2015). Study of the Design Optimization of AC-coupled Single-sided Silicon Strip Sensors. Korean Phys..

[4] Lee S.C., Jeon H.B., Kang K.H., Park H., Lee D.H., Lee M.W., Park K.S. (2019). Photo-Responses of Silicon Photodiodes with Different ARC Thicknesses for Scintillators. J. Korean Phys. Soc..

[5] Amptek, Inc. Datasheet Charge Sensitive Preamplifier and Shaping Amplifier A225. https://www.amptek.com/-/media/ametekamptek/documents/resources/specs/a225.eps?la=en&revision=718850f2-d24d-4d65-ba3f-040406c37b6e.

[6] Wigmans R. (2017). Calorimetry-Energy Measurements in Particle Physics.

[7] Ceccucci A. (1995). LKr calorimetry for the CP violation experiment NA48: Recent test beam results. Nucl. Instrum. Methods Phys. Res. Sect. A Accel. Spectrometers Detect. Assoc. Equip..

[8] Lee S.C., Jeon H.B., Kang K.H., Park H. (2016). Study of Silicon PIN Diode Responses to Low Energy Gamma-Rays. J. Korean Phys. Soc..

[9] Yan J., Liu R., Li C., Jiang L., Lu X.X., Zhu T.H. (2010). Energy calibration of a BC501A liquid scintillator using a *γ*-*γ* coincidence technique. Chinese Phys..

[10] Scherzinger J., Jebali R.A., Annand J.R.M., Fissum K.G., Hall-Wilton R., Kanaki K., Lundin M., Nilsson B., Perrey H., Rosborg A. (2016). The light-yield response of a NE-213 liquid-scintillator detector measured using 2–6 MeV tagged neutrons. Nucl. Instrum. Methods Phys. Res. Sect. A Accel. Spectrometers Detect. Assoc. Equip..

[11] Agostinelli S., Allison J., Amako K., Apostolakis J., Araujo H., Arce P., Asai M., Axen D., Banerjee S., Barrand G. (2003). GEANT4–a simulation toolkit. Nucl. Instrum. Methods Phys. Res. Sect. A Accel. Spectrometers Detect. Assoc. Equip..

[12] Kim K.R., Park B.S., Lee H.R., Kang K.S., Kang S.W., Choi B.H. 50 MeV proton beam test facility for low flux beam utilization studies of PEFP. Proceedings of the Agricultural Policy Advisory Committee (APAC).

[13] Knoll G.F. (2010). Radiation Detection and Measurement.

